# Direct Observation of Competing Prion Protein Fibril Populations with Distinct Structures and Kinetics

**DOI:** 10.1021/acsnano.2c12009

**Published:** 2023-02-20

**Authors:** Yuanzi Sun, Kezia Jack, Tiziana Ercolani, Daljit Sangar, Laszlo Hosszu, John Collinge, Jan Bieschke

**Affiliations:** † MRC Prion Unit at UCL/UCL Institute of Prion Diseases, University College London, London W1W 7FF, United Kingdom

**Keywords:** prion protein, protein misfolding, polymorphic amyloid structures, real-time kinetic measurements, super-resolution microscopy

## Abstract

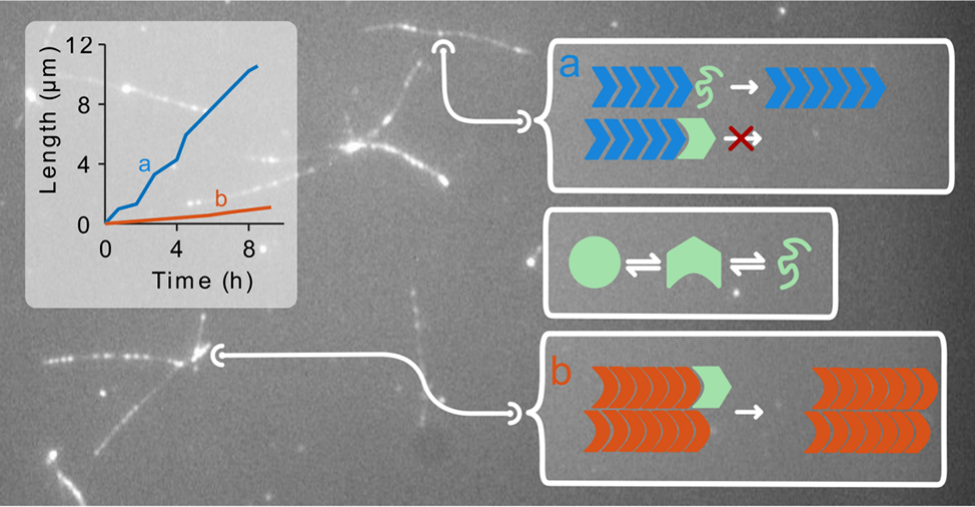

In prion diseases, fibrillar assemblies of misfolded prion protein (PrP) self-propagate by incorporating PrP monomers. These assemblies can evolve to adapt to changing environments and hosts, but the mechanism of prion evolution is poorly understood. We show that PrP fibrils exist as a population of competing conformers, which are selectively amplified under different conditions and can “mutate” during elongation. Prion replication therefore possesses the steps necessary for molecular evolution analogous to the quasispecies concept of genetic organisms. We monitored structure and growth of single PrP fibrils by total internal reflection and transient amyloid binding super-resolution microscopy and detected at least two main fibril populations, which emerged from seemingly homogeneous PrP seeds. All PrP fibrils elongated in a preferred direction by an intermittent “stop-and-go” mechanism, but each population possessed distinct elongation mechanisms that incorporated either unfolded or partially folded monomers. Elongation of RML and ME7 prion rods likewise exhibited distinct kinetic features. The discovery of polymorphic fibril populations growing in competition, which were previously hidden in ensemble measurements, suggests that prions and other amyloid replicating by prion-like mechanisms may represent quasispecies of structural isomorphs that can evolve to adapt to new hosts and conceivably could evade therapeutic intervention.

The abnormal aggregation of cellular proteins into insoluble amyloid is associated with a number of human diseases,^1^ including neurodegenerative disorders such as Alzheimer’s Disease, Parkinson’s Disease, and prion diseases.^2^ In prion diseases, benign cellular prion protein (PrP^C^) is converted to fibrillar assemblies of misfolded PrP, which self-propagate by recruiting other PrP monomers.^3,4^ This autocatalytic replication process defines the infectivity of prions.^5^ During the misfolding process, PrP converts from α-helix-rich folded monomers to amyloid fibrils comprising of stacked parallel in-register β-sheets,^6−8^ but how the structural conversion occurs is not well understood on a molecular level.

In amyloid formation via nucleated polymerization mechanisms with secondary processes (reviewed in refs (2, 9)), fibril elongation is central to the increase of fibril mass and generally is the fastest assembly step. Recombinant PrP^C^ readily forms amyloid fibrils under partially denaturing conditions *in vitro* and the elongation kinetics of this reaction have been systematically studied by seeding assays in bulk.^10−12^ Under such conditions, fibrils grow via the addition of unfolded monomers through a mechanism analogous to Michaelis–Menten enzyme kinetics.^10^ While natively folded PrP^C^ can inhibit elongation, natively unfolded protein does not.^10^ It has been hypothesized that prions exist as a “cloud” of conformers, and in which prions can evolve through conformational “mutation” and “selection”, analogous to the molecular principles of Darwinian evolution.^13−15^ However, bulk reactions cannot capture the variations in assembly of heterogeneous fibril structures, such as seen in prion strains, which allows prion strains to evolve and to adapt to different hosts.

Aβ fibrils formed *in vitro*(16,17) were shown to adopt different structures depending on incubating conditions. *Ex vivo* Aβ42 fibrils exhibited distinct structures with a direct link to the disease type.^18^ PrP fibrils formed *in vitro* showed a high level of polymorphism even under the same incubation condition.^19,20^ Structural polymorphism is also observed for authentic prions. Fibrils from different prion strains, which are associated with distinct clinical and neuropathological features,^21^ were shown to have distinct misfolded PrP structures.^3,6−8,22^

Emerging studies have used atomic force or optical microscopy to analyze fibril elongation kinetics for a range of other amyloidogenic proteins on a single-particle level.^23−28^ These studies revealed fine-grained growth properties such as stop-and-go patterns, growth polarity, and fibril breakage.^23−29^

## Results

We adapted total-internal-reflection microscopy (TIRFM) and transient amyloid binding (TAB) super-resolution microscopy to analyze fibril elongation rates of synthetically formed recombinant mouse PrP 91–231 (MoPrP 91) fibrils and authentic prion rods on a single-particle level. We systematically studied the effect of PrP concentration, PrP unfolding and temperature on elongation rates to understand fibril growth mechanisms. Analysis of the growth of single fibrils allowed us to test whether fibril populations are typically homogeneous or whether fibrils formed under seemingly homogeneous starting conditions can result in multiple fibril types with distinct fibril structures and different elongation mechanisms.

### Real-Time Kinetic Analysis of Single Fibril Growth

**Figure 1:**
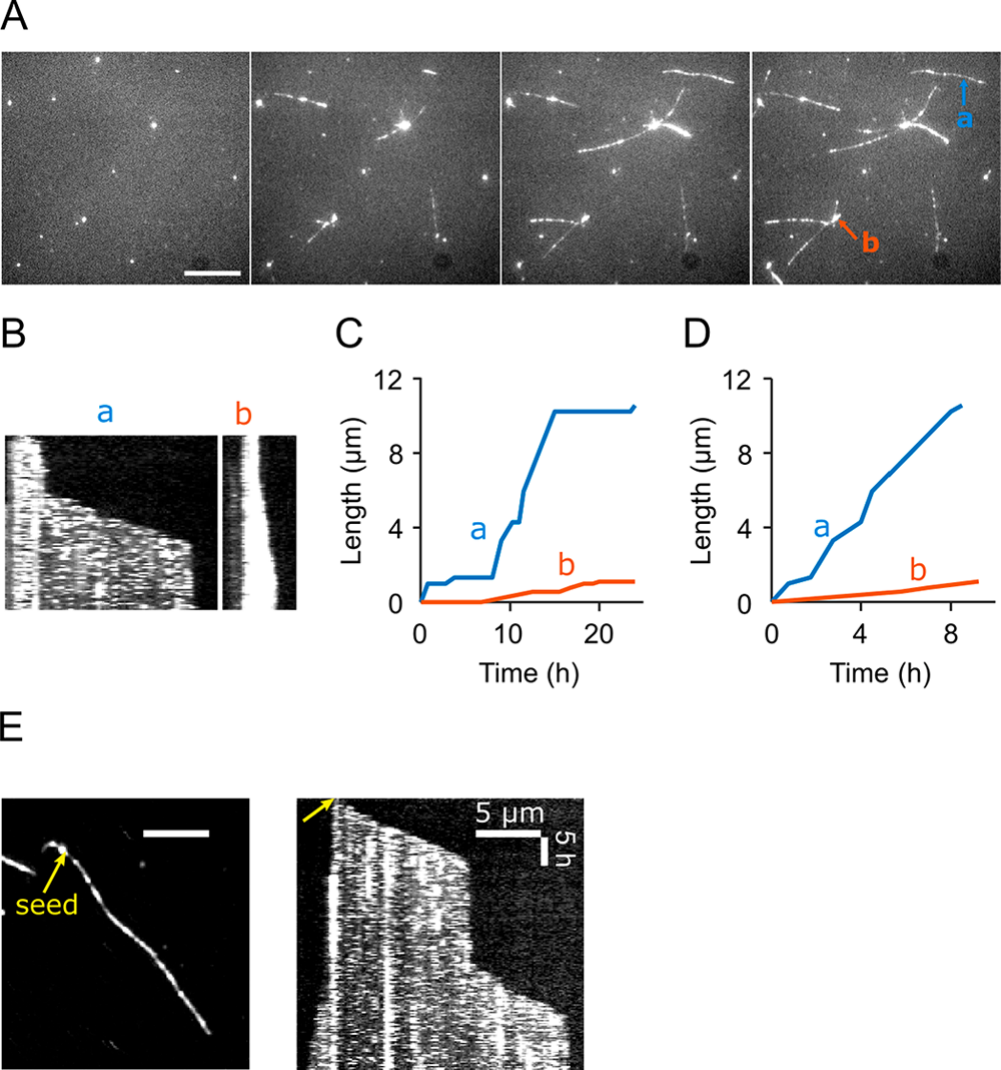
Real-time MoPrP 91 fibril elongation analysis observed by TIRFM. (A) Representative region imaged at multiple time points (0 h, 8 h, 16 h, and 24 h, respectively) showing the elongation from dot-like seeds into long, isolated fibrils or fibril clusters (conditions: 2 M GdnHCl, 1 μM MoPrP 91, 37 °C). The scale bar represents 10 μm. (B) Kymographs of two fibrils (a and b) indicated by blue and orange arrows in (A), respectively. The *x*-axis in each kymograph represents length along a line passing through the fibril axis, and *y*-axis corresponds to time. (C) Length versus time traces of fibril a (blue solid line) and b (orange dashed line) converted from fibril edges in kymographs. (D) Length versus time traces of fibrils a and b after removing stalls. (E) Left panel: the image of a fibril elongated for 47 h, generated by running Z-projection (projection type: standard deviation) command in ImageJ for a stack of 190 slices recording the growth of this fibril with 15 min interval. Arrow points to the original seed at *t* = 0 h. Right panel: kymograph of the fibril.

For elongation experiments, fibrillar MoPrP 91 seeds were generated through sequential seeding (Figure S1A,B) and deposited onto the bottom of an inverted microscope chamber. The unbound seed was removed and fibril elongation was monitored by TIRFM over a range of PrP^C^ concentrations (0.3–10 μM), GdnHCl concentrations (1.4–2.3 M) and temperatures (27–40 °C) in the presence of Nile Blue (400 nM) to image PrP amyloid. Nile Blue displays greatly enhanced fluorescence emission upon amyloid binding but lacks the increase in Stokes shift of Thioflavin T (Figure S1G,H). Nile Blue and the closely related dye Nile Red have been used in super-resolution imaging of amyloid fibrils^30,31^ and lipid membranes.^32^Figure 1A and Movies MS1 and MS2 show seed elongation when using 2 M GdnHCl, 1 μM MoPrP 91 at 37 °C. Dot-like seeds grew gradually into long single fibrils or multiple fibrils in different directions, indicating seed clusters. A kymograph was generated for each fibril (Figure 1B) and converted to a fibril growth plot (Figure 1C).

Growth of individual fibrils was interrupted by stall phases in a “stop-and-go” growth. This growth pattern had previously been observed in other amyloid fibrils^25,26^ and was attributed to structurally incorrectly bound monomers/oligomers to the fibril ends, which have to detach or convert to the fibril fold before other molecules can add.^33^ We calculated the pause-free growth rates (Figure 1D) and the relative stall time percentages for further analysis. Strikingly, growth rates were mostly constant for individual fibrils, however, they could vary dramatically between different fibrils, as shown for fibrils ‘a’ and ‘b’ in Figure 1A–D. Fibril growth was highly directional (Figures 1A,E and Movies MS1 and MS2).

**Figure 2:**
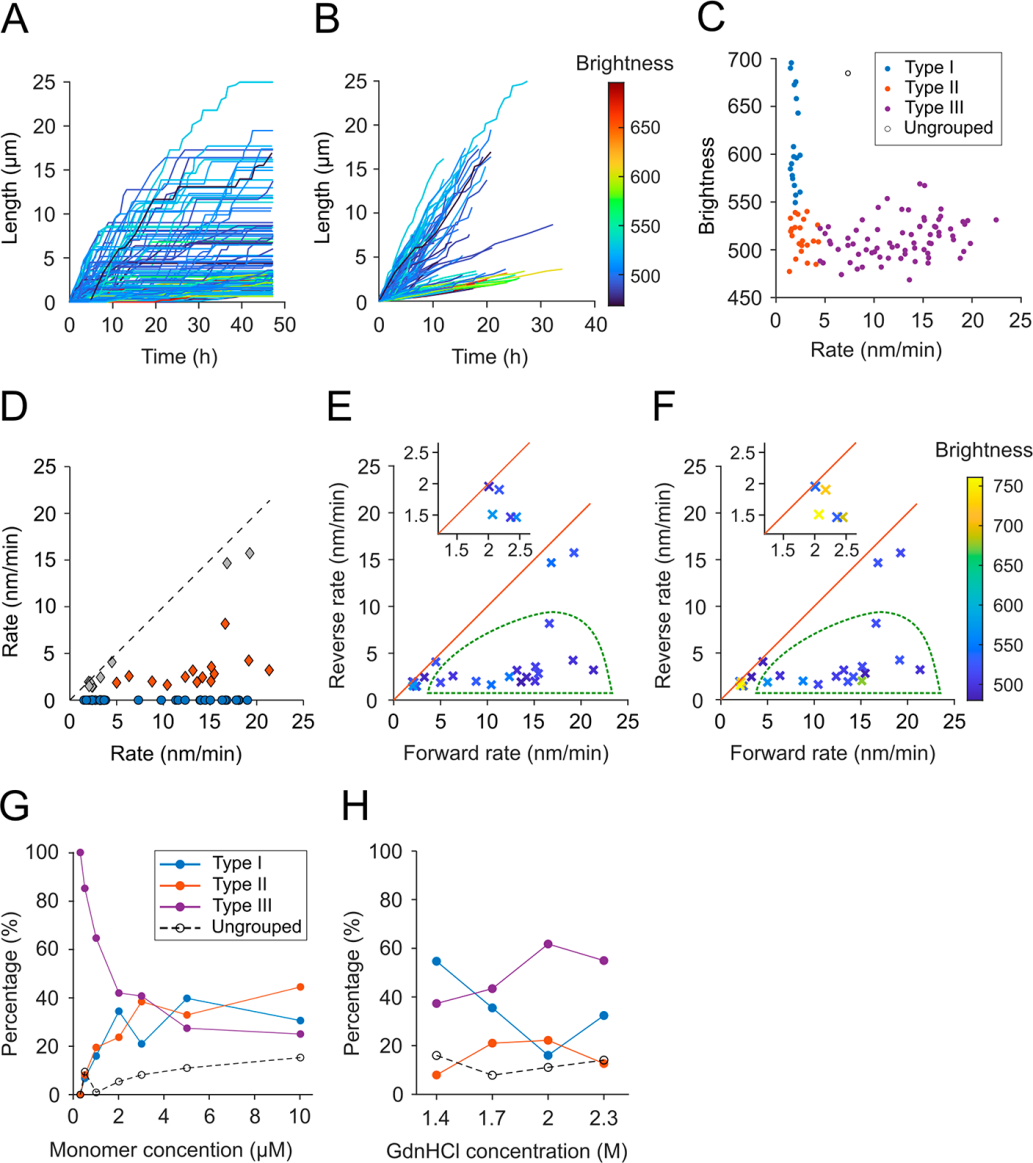
Kinetic analysis revealed multiple fibril populations. (A) Length versus time traces for fibrils elongated in 2 M GdnHCl with 1 μM PrP^C^ at 37 °C; traces are color-coded by fibril brightness. (B) Length versus time traces with stall phase removed. (C) Scatter plot of fibril’s brightness versus pause-free rate. Fibrils were grouped based on the clustering within the scatter plot: slow-growing and bright fibrils as type I (blue), slow, dim fibrils as type II (orange), and fast, dim fibrils as type III (purple). (D) Scatter plot of pause-free rates of single fibrils showing growth directionality. Blue circles on *x*-axis represent unidirectional fibrils; diamonds (red or gray) represent bidirectional fibril growth with the rate of its faster end on *x*-axis and the slower end on *y*-axis. (E) Scatter plot of bidirectional fibrils’ pause-free rates of opposing tips, color-coded by the brightness of the forward ends. The inset shows a zoomed-in view of the slow-rate regime. (F) Scatter plot as in (E), with each dot color-coded by the brightness of the reverse ends. (G) Dependence of three fibril types’ fractions on PrP^C^ concentration. (H) Dependence of fibril fractions on GdnHCl concentration. Analysis of five fields of view yielded standard deviations of 12%, 13% and 14% for type I, II, and III fibrils, respectively, at 1 μM PrP^C^, 2 M GdnHCl.

For a quantitative analysis of elongation kinetics, 114 independent fibril growth traces were analyzed as described above (Figure 2A,B). Strikingly, fibril growth rates did not fall into a single distribution as had previously been reported for other amyloidogenic proteins,^26^ even though initial seeds originated from sequential bulk seeding assays and formed a seemingly homogeneous population (Figure S1A,B). Growth traces in Figure 2A and B are color-coded by fibril brightness, showing that growth rates could be grouped into two clusters exhibiting slow or fast growth and distinct fluorescence intensities. A scatter plot of fibril brightness versus pause-free rate (Figure 2C) revealed the presence of three distinct types of fibrils: slow-growing bright fibrils (denoted as type I fibrils, blue), slow-growing dim fibrils (type II, orange), and fast-growing dim fibrils (type III, purple), and a small portion that did not fall into any group or displayed inconsistent growth rates (open circles). Hence, while bright fibrils were exclusively slow-growing, all fast fibrils were relatively dim. That these differences likely result from different fibril structures will be discussed below.

Figure 2D plots pause-free rates of fast (forward) versus reverse growth in a scatter plot with each dot representing the growth pattern of a single fibril. Most fibrils grew in one direction only (blue dots) or showed reverse growth being much slower than forward elongation (red diamonds). These data suggest that fibrils are structurally distinct at opposing ends, leading to easier and faster incorporation of PrP at one end over the other. Indeed, recent atomic models of PrP amyloid fibrils^34^ and prion rods^6,7^ feature a step pattern at the fibril end, resulting in two asymmetric growth surfaces. A small portion of fibrils, marked by gray diamonds, elongated at similar rates in both directions. Since light microscopy cannot resolve single seeds and seed clusters, these fibrils were likely seeded by clusters that happened to be aligned in their fibril growth axes.

We more closely examined the kinetics of fibrils displaying bidirectional growth (Figure 2E,F). Here, each bidirectional fibril is color-coded by the brightness of the forward end (E) and the reverse growing end (F), respectively. As indicated above, most bidirectional fibrils have a fast and a slow end (Figure 2D, red diamonds, green dashed area in Figure 2E,F). None of them falls into the “type I” category of slow, bright fibrils, but rather shows one fast growing dim end (type III) and one slow growing dim end (type II). This observation suggests that type II and III fibrils likely represent the same fibril structure elongating from the reverse or forward ends, respectively.

Overall, our methodology allowed us to analyze in large data sets how the environment altered stall percentage, growth rate, and partitioning between fibril populations.

### Elongation of Competing PrP Fibril Populations

Monomer concentration determined relative populations of type I, II, and III fibrils (Figure 2G). Type III fibrils were favored at lower monomer concentrations, accounting for over 80% below 1 μM, while high PrP concentrations shifted the equilibrium toward slower-growing type I and II fibrils.

To test how the unfolding of substrate PrP affected the fibril populations, elongation experiments were performed at GdnHCl concentrations ranging from 1.4 to 2.3 M, corresponding to a fraction of 0.8–52% unfolded protein under assay conditions (Figure S1C,D). Figure 2H shows that low denaturant concentrations favored the formation of slow-growing type I fibrils. Their population decreased with GdnHCl concentration, while the population of fast-growing type III fibrils increased. It should be noted that fast-growing fibrils occasionally detached from the surface at 2.3 M GdnHCl. Those fibrils were eliminated from rate analysis, which artificially depressed the fraction of type III fibrils under these conditions. To summarize, an increasing fraction of unfolded PrP monomer shifted the equilibrium between competing fibril seeds toward fast-growing, type III fibrils.

### PrP Fibrils with Different Kinetic Profiles Correspond to Distinct Structures

Next, we tested whether fibril types corresponded to distinct fibril structures by choosing reaction conditions, which favored the growth of type I or type III fibrils, respectively: (a) 1 M GdnHCl with 1 μM monomer (∼70% type I fibrils) and (b) 2 M GdnHCl with 1 μM monomer (∼60% type III fibrils).

**Figure 3:**
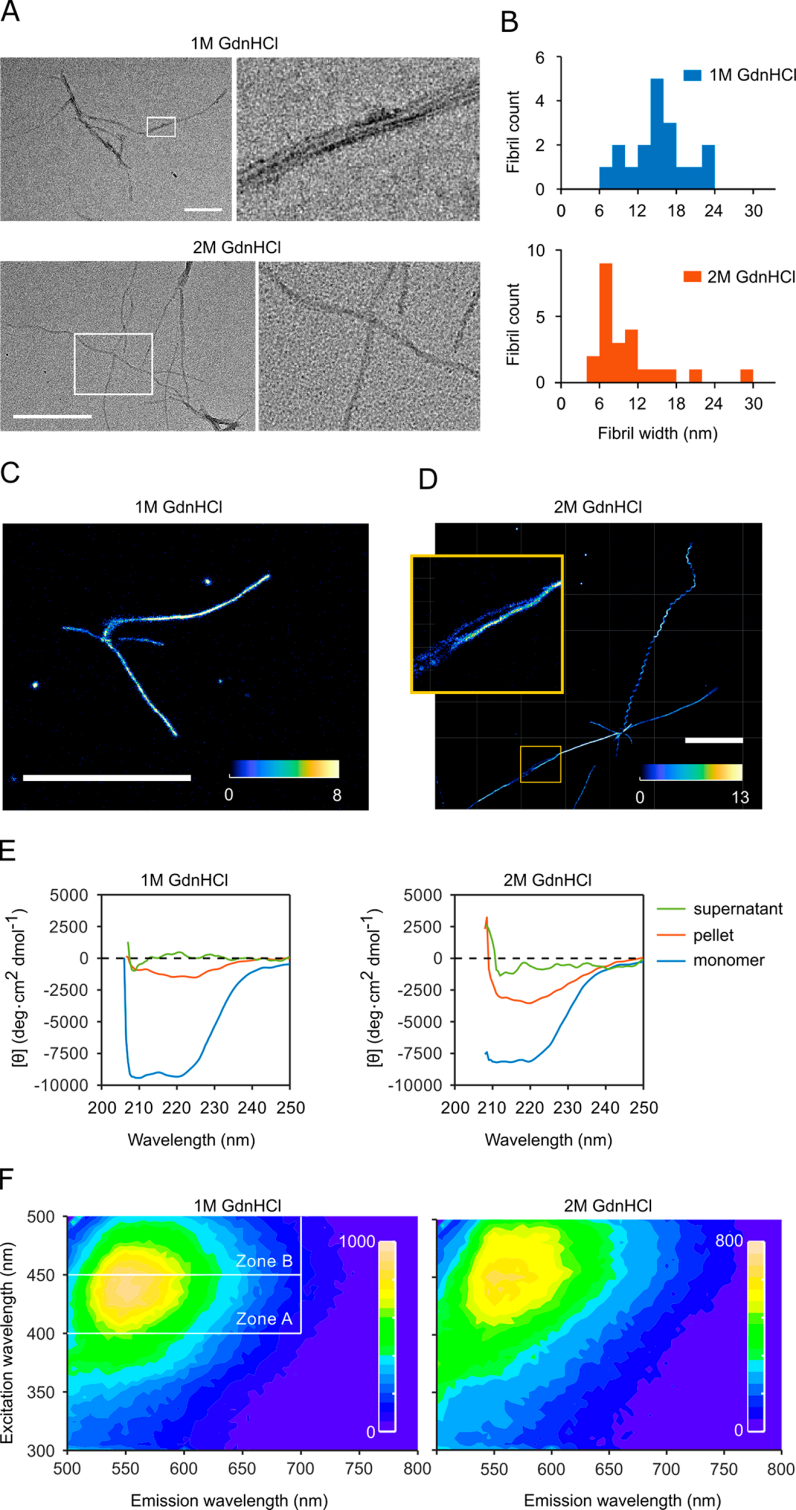
PrP fibrils with different kinetic profiles correspond to distinct structures. (A) Typical TEM images of fibrils elongated in 1 M GdnHCl (top) or 2 M GdnHCl (bottom); scale bar is 500 nm. (B) Width distribution of fibrils grown in 1 M GdnHCl (top) or 2 M GdnHCl (bottom). (C) Typical TAB image of fibrils elongated in 1 M GdnHCl. Images were acquired in the presence of 50 nM Nile Red. Brightness represents the number of localizations identified in each pixel; scale bar is 5 μm. (D) TAB image of fibrils elongated in 2 M GdnHCl and a zoomed-in view showing the branching of a fibril. (E) CD spectra of PrP^C^ or PrP aggregated in buffer with 1 M (left) or 2 M (right) GdnHCl. For aggregated PrP, ultracentrifugation was performed; supernatants and resuspended pellets were measured by CD separately. (F) Spectral fingerprinting contour maps of fibrils generated by sequential seeding in buffer containing 1 M GdnHCl (left) or 2 M GdnHCl (right). The partition of zones A and B is shown in the left panel.

We performed elongation experiments *in situ* on electron microscopy grids, imaged the elongated fibrils by EM and analyzed fibril diameters under both conditions (Figure 3A,B). We only analyzed fibril segments that were longer than 500 nm to exclude initial seed fibrils (Figure S1B). PrP fibrils grown under the two conditions had distinct distributions of fibril widths: 16–18 nm wide fibrils were dominant in condition (a) favoring type I fibrils, while condition (b) favored type III fibrils generated mostly 6–8 nm wide fibrils. As can be seen in Figure 3A and B, the two different fibril widths likely represent double-strand and single-strand fibrils, respectively. Single-strand fibrils observed under condition (b) tended to be much longer than fibrils under condition (a) reflecting faster fibril growth.

To confirm this conclusion, fibrils generated under conditions (a) and (b) were analyzed by transient amyloid binding (TAB) microscopy (Figures 3C,D and S2A).^35^ The colors represent the number of individual dye molecule localizations, i.e., “brightness”. Although amyloid fibrils are too narrow for single and double-strand fibrils to be directly resolved, two distinct populations of fibrils are clearly apparent. One group includes straight bright (type I) fibrils, which unspliced occasionally (Figure 3D, inset) revealing their multistrand structure. In contrast, TAB and EM both resolved single strand with curvy morphologies for type III fibrils.

Interestingly, both EM and TAB imaging identified additional fibril morphologies, albeit at low abundance. We observed single-strand straight fibrils, single-strand curved fibrils, multiple-strand fibrils with regular twisting (Figure S2B), multiple-strand fibrils without twisting and helically coiled fibrils (Figure S2A–C). Regrettably, low abundance and detachment of helical fibrils from the glass surface prevented the systematic analysis of elongation kinetics for these fibril types. The variety of fibril morphologies suggests that PrP fibrils, rather than being a single, homogeneous species, can adopt multiple amyloid structures, likely the result of subtly different underlying folds of the peptide chain. These fibril isomorphs were missed by the lack of spatial resolution in TIRF microscopy.

EM and TAB images suggest that fibril types may not only differ in their number of strands but also in their internal architecture. To substantiate this hypothesis, the secondary structures of fibrils formed under both conditions were studied by circular dichroism (CD) spectroscopy (Figure 3E). Two rounds of seeding in solution were performed for conditions (a) and (b) to enrich type I and type III fibrils, respectively. Under both conditions, the spectra of the pelleted and resuspended fibrils had minima between 218 and 225 nm, indicating the presence of β-sheets. However, spectra were distinct with minima at ∼220 for type III, and at ∼225 nm for type I enriched fibrils, respectively, confirming that fibrils generated under the two conditions had distinctly different secondary structures.

**Figure 4:**
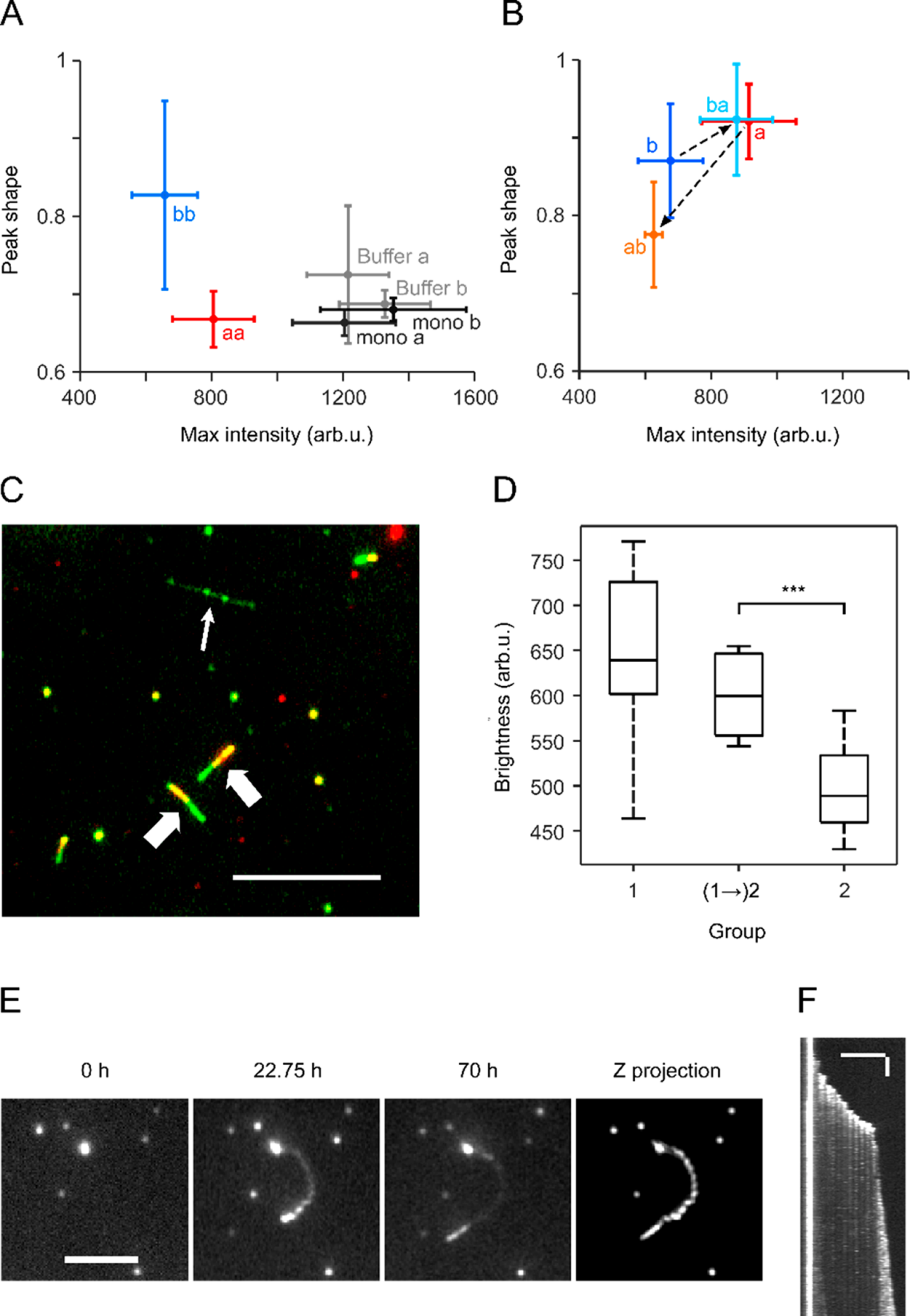
Fibril types propagate faithfully under competing conditions. (A) Plot of peak shape (integral of zone B/integral of zone A) against maximum intensity for samples aa and bb, which were seeded twice in the corresponding buffer, and for buffer and monomer samples in the corresponding buffer a and b. (B) Comparison of peak shape versus maximum intensity for samples a, ab, b, and ba. (C) Composite image of an ROI of a two-phase seed elongation experiment showing seeds elongated in buffer with 1 M GdnHCl for 71 h (red channel) and then switched to 2 M GdnHCl (green channel) for 41.5 h. PrP^C^ concentration was 1 μM in both phases. The thin arrow refers to a fibril that only grew in the second condition; wide arrows point to fibrils which elongated in both conditions. The scale bar represents 10 μm. (D) Brightness analysis for fibril segments that grew in the first seeding phase (group 1), fibril segments that grew in the second seeding phase from an elongated fibril in the first phase (group (1 → 2), and fibrils that exclusively grew in the second phase (group 2). Nineteen fibrils in total were analyzed. (E) Change of fibril type during elongation. Panels show the fibril imaged at 0 h, 22.75 h, 70 h and a Z-projection of the growth stack from 0 to 70 h. The scale bar represents 5 μm. The experiment was conducted in a buffer containing 1.4 M GdnHCl and 1 μM PrP at 37 °C. (F) Kymograph of the fibril shown in (E). The horizontal scale bar represents 5 μm, and the vertical scale bar represents 5 h.

This interpretation was confirmed by spectral fingerprinting of the luminescent conjugated oligothiophene dye (LCO) dye heptamer formyl thiophene acetic acid (h-FTAA). LCO dyes display spectral shifts upon binding to amyloid fibrils, which can differentiate structural subtypes of amyloid.^36^Figure 3F shows 3D excitation–emission contour plots of h-FTAA bound to PrP fibrils grown under conditions (a) and (b) from seeds also made under the same condition. These fibrils are denoted ‘aa’ and ‘bb’, respectively, in Figure 4A. Their spectra differ both in peak shape as defined by the ratio B/A of fluorescence emission integrated over zones A and B, respectively, and in their maximal fluorescence intensities (Figures 3F and 4A).

Lastly, we compared their resistance to digestion by Proteinase K (PK), by depositing PrP aggregates grown in 1 or 2 M GdnHCl, respectively, on a glass slide and imaging PrP aggregates pre- and post digestion (Figure S3). While fibrils grown under both conditions were partially resistant to PK-digestion, PrP aggregates grown under conditions that enrich type III fibrils showed a significant (*p* < 10^–8^) loss of fluorescence intensity when compared to aggregates grown under conditions favoring type I fibrils (Figure S3C,D). CD spectroscopy, spectral fingerprinting, and PK digestion, therefore, all support that fibril types I and III not only correspond to double- and single-strand fibrils, respectively, but that both types of fibrils feature different internal structures.

### Elongation Fidelity

We next determined whether fibril types could faithfully propagate their respective structures under adverse conditions, which favor another fibril type, or whether, alternatively, fibril types were determined only by solution conditions. Here, seeds grown in condition (a) favoring type I fibrils were elongated in condition (b) favoring type III fibrils and vice versa. When fibrils were grown in solution and analyzed in bulk, second-generation fibrils first grown in condition (b) then in (a) (Figure 4B; ‘ba’) had similar spectral fingerprints to first-generation seeds ‘a’. Likewise, the footprint of first-generation ‘a’ seeds shifted when elongated under (b) conditions (Figure 4B, ‘ab’). This result seemed to suggest that solution conditions rather than seed template determined fibril structure. However, a closer analysis of single fibril growth revealed this not to be the case.

To probe whether fidelity to a template depends on seed type or solution composition, or on some combination of both of these, we designed a two-phase experiment. In the first phase fibrils grew from seeds under buffer condition (a) favoring the formation of type I fibrils. Then the buffer was switched to condition (b) and fibril growth was monitored for a further 88 h by TIRFM. In Figure 4C, fibrils present at the beginning of the second phase are shown in red, and fibrils grown or elongated during the second phase are shown in green. Correspondingly, fibrils from the first phase appear yellow, when still present in the second phase of the experiment, while red-stained dots correspond to initial seeds that had detached during the second phase.

In the second elongation phase, type I fibrils continued to grow (Figure 4C, wide arrows); type III fibrils started to grow from dot-like seeds that had not extended in phase 1 or from a position where no obvious seed was observed (Figure 4B, thin arrow). Figure 4D plots the brightness profiles of fibril segments grown in the first and second phases. Fibril brightness did not significantly change on elongation after the buffer exchange (Figure 4D, groups 1 and (1 → 2). These data indicate that elongating fibrils retained their fibril structures after the switch of buffer conditions and that type III fibrils were not cross-seeded by type I and vice versa. Our data, therefore, suggest that buffer conditions shift the equilibrium between fibril types, so that type I fibrils could outcompete type III in condition (a) and vice versa, as shown in Figure 4B, but that type and structure of every single fibril was specifically templated by the seed type rather than determined by buffer conditions.

Although most fibrils elongated faithfully, in rare cases fibril types switched during growth. Figure 4E shows a helical fibril similar to Figure 3D, which switches to a type I fibril at ∼23 h of growth. The switch in fibril morphology coincides with a drop in elongation rate typical for type I fibrils (Figure 4F). While we would expect the environment to affect the frequency of these “mutation” events, they only occurred in <1% of fibrils and thus were too rare for systematic analysis.

### Competing PrP Fibril Populations Elongate with Distinct Mechanisms

**Figure 5:**
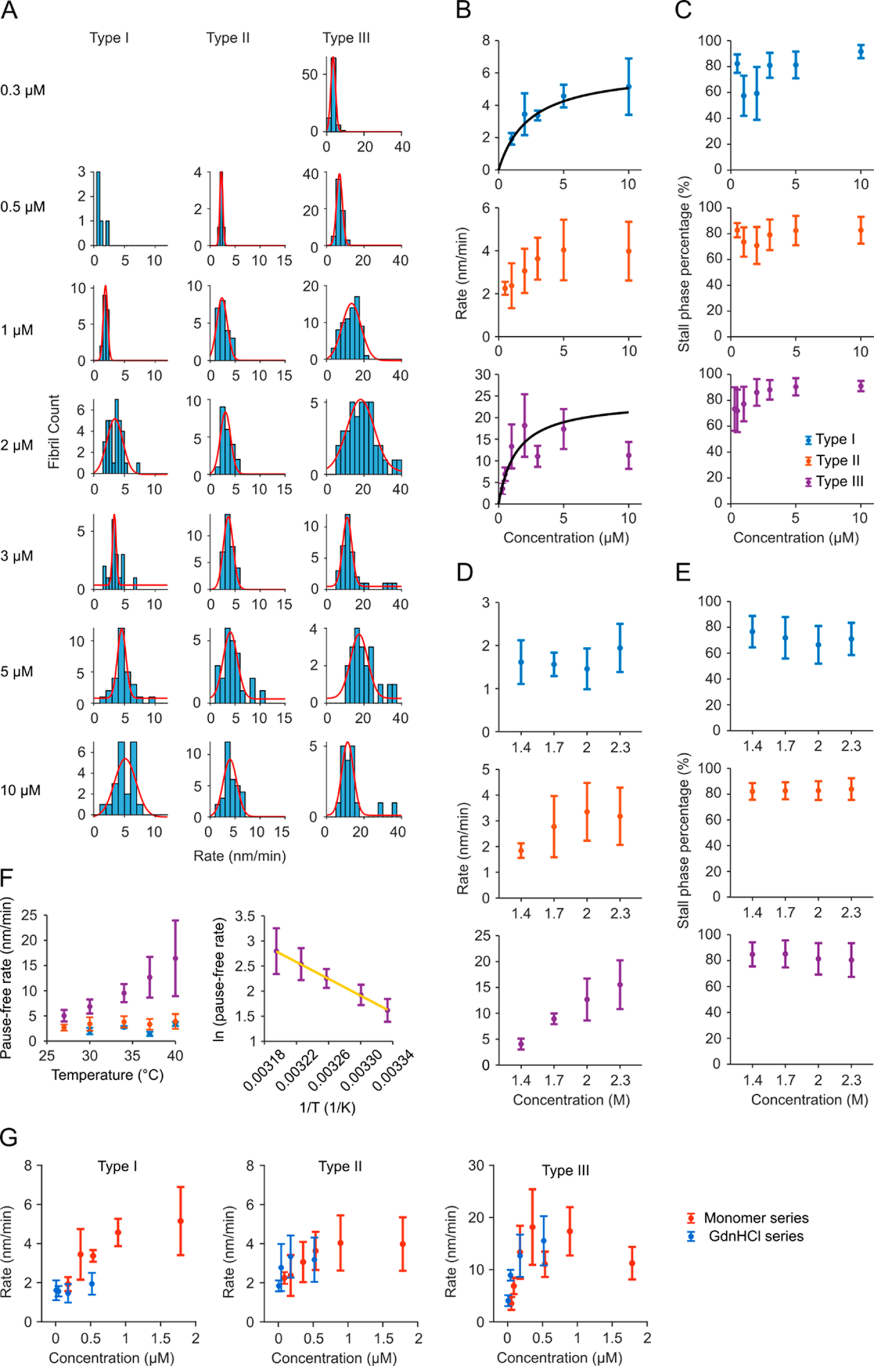
Kinetic analysis of individual fibril types under different conditions. (A) Pause-free rate distribution of three types of fibrils in monomer concentration series. Red curves represent Gaussian fitting to the distribution. (B) Dependence of pause-free rates of type I (top), type II (middle), or type III (bottom) fibrils on PrP^C^ concentration. Pause-free rates are the peak positions taken from Gaussian fittings in (A), and error bars represent σ. The dashed curves in the top and bottom panels represent fit of a Michaelis–Menten type mechanism to the data. In the bottom panel data points at 3 μM and 10 μM were excluded from the fit. (C) Dependence of stall phase percentages of type I, II, or III fibrils on PrP^C^ concentration; stall percentages are the average value of fibrils of the specific type. Errors are standard deviations. (D) Dependence of pause-free rates of type I (top), II (middle), or type III fibrils (bottom) on GdnHCl concentration. (E) Dependence of stall phase percentages of type I, II, or III fibrils on GdnHCl concentration. (F) Temperature dependence of fibril pause-free rate. Left: pause-free rates of type I (blue), II (orange), or III (purple) fibrils under different temperatures. Right: Arrhenius plot for type III fibrils. The straight line is a linear fit of the five data points. (G) Dependence of pause-free rate of three fibril types on unfolded monomer concentration in PrP^C^ concentration series (red) and GdnHCl concentration series (blue). See also Figures S4–S6 for fibril distribution histograms.

Specific templating of fibril types suggests the presence of distinct elongation mechanisms. To test this hypothesis, we analyzed the dependence of fibril elongation on PrP monomer concentration, denaturant concentration, and temperature. Figures 5A and S4B show the distributions of stall-free growth rates and stall percentages for fibrils grown at 0.3, 0.5, 1, 2, 3, 5, and 10 μM monomer concentration, respectively, separated by fibril types (Figure S4C), while Figure S4A represents the data for the entire fibril population. Type III fibrils exhibited a strong concentration dependence at low PrP concentrations with inhibition at high monomer concentrations, which was not seen for Type I and II fibrils (Figure 5B). Type I fibrils feature broad distributions of stall percentages with an average of (57 ± 16) % and (59 ± 20) % at 1 μM and 2 μM PrP concentrations, respectively (Figures 5C and S4B,D), whereas type III fibrils show stall percentages of (77 ± 13) % and (86 ± 10) % at 1 μM and 2 μM PrP, which are significantly higher (*p* < 0.0001) and more narrowly distributed than those of type I fibrils.

Below, we show that different steps of the assembly mechanism were rate limiting for fibril types I and III. However, the large variance for type II fibrils’ pause-free rates made it difficult to derive a mechanism for them.

Fast-growing type III fibrils saw a nearly linear concentration dependence between 0.3–2 μM, while higher PrP concentrations inhibited fibril growth. In contrast, bright, slow type I fibrils exhibited a weaker concentration dependence and did not show inhibition at high PrP concentrations. In the simplest case, fibril elongation could be described as catalytic conversion of monomer (M) to fibril units (F) at the end of a growing fibril (E) in which the successful conversion of the monomer yields a fresh catalytic interface for further fibril growth (eq 1). This situation is analogous to a two-step Michaelis–Menten-type reaction scheme (eq 2) involving initial binding and subsequent structural rearrangement:
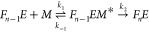
1or
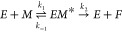
2

We have analyzed elongation rates under the assumption of excess monomer to calculate *K*_m_ = (k_–1_ + *k*_2_) /*k*_1_ and *k*_2_, respectively (line graphs in Figure 5B). This simple model fits the concentration dependence of type I fibrils with *K*_m_ = 2.4 ± 0.3 μM, and *k*_2_ = 0.44 ± 0.05 s^–1^, assuming type I fibrils were made of double strands. It could adequately describe type III fibril growth at low to medium monomer concentrations (*K*_m_ = 1.4 ± 0.5 μM, *k*_2_ = 0.8 ± 0.2 s^–1^), but failed to capture the inhibition of type III fibril growth at higher monomer concentrations. Noncompetitive inhibition by unproductive binding of PrP monomers could account for this inhibition.^10^ Alternatively, PrP could be forming off-pathway assemblies at high protein concentrations, which bind at the fibril end and prevent fibril growth.

A bimolecular reaction model could be assumed for type III fibrils in the linear phase (0.3–1 μM) when structural conversion is not rate limiting, yielding a rate constant of (4.5 ± 0.2) × 10^5^ M^–1^ s^–1^. This value is comparable to elongation rate constants of multiple amyloid fibrils in literature^33^ and is well below the diffusion limit.

Qualitatively, the concentration dependence of type II fibrils’ growth rates resemble that of type I fibrils, with an initial increase with PrP concentration followed by saturation without obvious inhibition at high PrP concentrations. This might indicate a similar growth mechanism involved for the two slow-growing fibrils despite their differences in fibril brightness.

To further analyze the assembly mechanism, we determined the dependence of fibril growth rates on denaturant concentration from 1.4–2.3 M GdnHCl at a fixed PrP concentration (Figures 5D,E and S5). Under our reaction conditions, elongation rates of type III fibrils increased linearly with GdnHCl concentration, suggesting that this fibril type grows by incorporating unfolded PrP molecules. Indeed, data from GdnHCl and monomer concentration series fell into one graph when plotting elongation rate against unfolded monomer concentration (Figure 5G). In contrast, type I fibril elongation showed no dependence on GdnHCl concentration. Concentration dependence of type II fibrils was weak and laid within experimental error. This behavior is compatible with the incorporation of a partially folded intermediate, whose concentration changes weakly in the GdnHCl range probed in the experiment, or with slow conformational change being rate-limiting.

Stall percentages increased with PrP monomer concentration for all three fibril types (Figures 5C and S4B). While stall percentage was low (57 ± 16) % for type I fibrils at 1 μM PrP, it increased markedly to (92 ± 5) % at 10 μM monomer concentration. Type II and III fibril stalling increased from (74 ± 11) % to (82 ± 10) % and (77 ± 13) % to (91 ± 4) %, respectively, indicating that stall events dominated at high PrP concentrations. Both, fibril stalling and the drop in elongation rate at high monomer concentrations observed for type III fibrils, indicate a concentration-dependent negative feedback loop, which inhibits fibril growth. While both processes appear to operate in different time scales, the distinction is somewhat arbitrary, because it depends on the sampling rate of our kinetic assay with stall events <15 min being perceived as a reduction in elongation rate. Both stalling and slowed elongation are most likely due to PrP bound to the fibril ends, either in the form of monomers or nonfibrillar assemblies, which could not convert into the amyloid fold. It is possible that, despite the presence of GdnHCl, high PrP concentrations facilitated the formation of nonfibrillar PrP assemblies that could contribute to the stalling of fibril growth. However, GdnHCl concentration did not change the stall percentages for any of the three fibril types, arguing against this interpretation.

Elongation rates were measured at five temperatures (27, 30, 34, 37, and 40 °C) at fixed solution conditions (2 M GdnHCl, 1 μM PrP^C^) to determine the activation energies for elongation of the three fibril types (Figures 5F and S6). The fraction of unfolded protein varied from 5% at 27 °C to 40% at 40 °C under these conditions (Figure S1E,F). Pause-free elongation rates for type III fibrils had a strong temperature dependence, which followed the Arrhenius equation with activation energy (*E*_a_) of 70 ± 2 kJ/mol. This is comparable to the literature value of ∼50 kJ/mol under conditions when unfolded monomer is abundant.^11^ In contrast, pause-free rates for type I and II fibrils did not change with temperature, suggesting the rate-limiting step was not temperature-dependent. This may be due to entropic and enthalpic contributions to the activation energy largely canceling each other out.^37^

In summary, our single-fibril kinetic analysis indicates that at least two different PrP fibril types, I and III, were growing in competition from a seemingly homogeneous pool of seeds. While growth conditions could shift the equilibrium between fibril types, fibrils mostly elongated faithfully without apparent cross-seeding and by distinct mechanisms, similar to what has been posited for prion strains. Our data suggest the presence of a polymorphic “cloud” of fibril conformations, which compete for the same substrate pool, raising the question of whether elongation of authentic prion seeds shares the same structural and mechanistic diversity.

### Elongation of Authentic Prion Seeds

We analyzed the elongation of seeds from two mouse prion strains, RML and ME7, in the presence of recombinant monomer to probe how their elongation kinetics and fibril structures differ from synthetic fibril seeds and between different prion strains.

**Figure 6:**
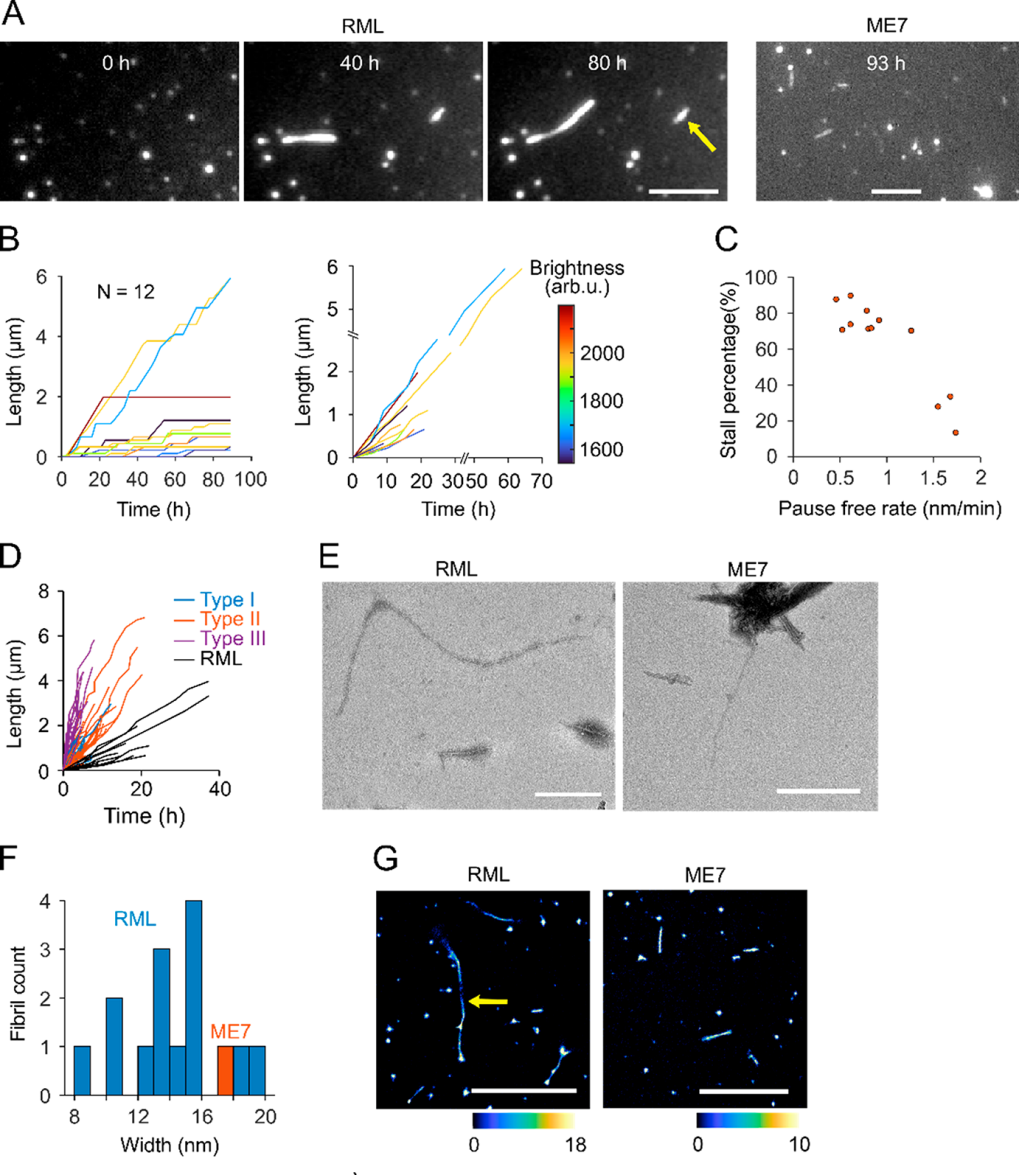
Elongation of prion rods. (A) TIRFM images showing the elongation of RML and end point of ME7 fibrils. The scale bar represents 5 μm. (B) Length versus time traces for 12 RML fibrils with stall phases included (left) or removed (right). Traces are color-coded by fibril brightness. (C) Stall percentages of each fibril plotted against the brightness. Stall percentage was calculated using the time before the last stall phase as total time. (D) Length versus time traces with stall removed of RML overlaid with grouped elongation traces of recombinant seeds. (E) TEM images of RML (left) or ME7 (right) fibrils elongated on EM grids. The scale bar represents 500 nm. (F) Width distribution of RML fibrils and an ME7 fibril elongated on EM grids. (G) TAB images of elongated RML fibrils. Scale bar 5 μm. Arrows in panels A and G indicate bidirectional growth.

RML and ME7 prion rods were purified from infected mouse brain as previously described,^38,39^ adsorbed onto coverslips and incubated with MoPrP 91 monomer (10 μM) in 2 M GdnHCl. RML seeds readily elongated under these conditions (Figure 6A). In contrast, only few short fibrils grew from ME7 seeds. A quantitative analysis was performed for *n* = 12 RML-seeded fibrils, and the growth traces and stall percentages are shown in Figure 6B and C, respectively. Similar to the fibrils grown from synthetic seeds, pause-free rates within a fibril were nearly constant. However, fibrils displayed diversity in growth rates, stall percentages and fibril brightness (Figure 6B,C). Elongation rates of RML seed (0.4–1.7 nm/min) were generally slower than all three types of synthetic seeds grown under the same condition (Figure 6D). However, unlike recombinant seed elongation, rates and brightness of RML seeded fibrils did not correlate, so no distinct fibril types could be identified.

*In situ* elongation experiments with RML and ME7 prion rods on EM grids confirmed that RML prion rods readily elongated into slightly curved fibrils with an average width of 12–16 nm (Figure 6E,F). In contrast, the one fibril that could clearly be identified as having been newly seeded from an ME7 prion rod was a short, single-strand fibril with a diameter of ∼18 nm. Similar to EM, TAB microscopy showed long fibrils growing from RML seeds, while ME7 yielded short, straight fibrils (Figure 6G). Intriguingly, fibrils grown from RML and ME7 prion rods displayed repeating intensity patterns with a period of 134 ± 4 nm and 153 ± 8 nm, respectively (Figure S7). These periods coincide with the crossover distances of RML and ME7 prion fibrils recently determined by cryo-EM,^6−8^ suggesting that elongation of prion rods preserves elements of prion fibril architecture.

Overall, our data suggest that both RML and ME7 rods are capable of elongating under the conditions examined here, but suggests that growth rates are starkly different between the two strains. The elongation rates of RML-seeded fibrils are slow compared to synthetic fibril seeds. A diversity of fibril brightness and elongation rates suggest that the purified RML prion samples can template more than one fibril structure. Whether this points to a lack of fidelity in templating their structure under denaturing conditions or whether it indicates the presence of structural heterogeneity of a prion quasispecies will have to be resolved by future kinetic studies under physiological replication conditions.

## Discussion

Distinct fibril structures, which can replicate by templated monomer addition, are thought to be the structural basis for prion strains. Cryo-EM of three prion strains, RML,^6^ ME7,^8^ and 263 K,^7^ albeit propagated in two different species, displayed homogeneous structures with similarities in their overall arrangement of the polypeptide chain, which nevertheless substantially alter fibril morphology and charge patterning. Distinct structures of patient-derived amyloid corresponded to specific tauopathy phenotypes.^40−43^ Rapid progressing AD seeded distinct fibril structures from normal sporadic AD,^44^ and Aβ42 fibrils that were isolated from patients suffering from sporadic and familial AD showed two distinct morphologies.^18^ Notably, fibril structures in each patient, while not entirely homogeneous, were dominated by one isomorph, suggesting competing templating processes within the affected patient brain.^18^

Structural isomorphs were also observed within homogeneous amyloid preparations.^45,46^*De novo* aggregation of hamster PrP 90–231^19,20^ yielded distinct aggregation kinetics and morphologically different fibrils under the same conditions, which suggested the formation of structurally different nuclei and different folding pathways of PrP. Our work showed that different fibrils can form and coexist in one sample, and characterized their distinct growth properties and mechanisms using single-particle approaches.

Our single-molecule measurements on PrP seeded elongation using TIRFM revealed at least two main groups of fibrils based on their brightness and kinetic profile (Figure 2), with fast-growing, dim single strand fibrils and slow-growing and bright double-strand fibrils. CD and spectral fingerprinting indicated that these types had distinct structural arrangements in the fibril (Figure 3). Thus, fibrils displayed distinct dynamic properties, which were linked to their specific structures. Interestingly, most fibrils seeded by RML and ME7 prion rods had diameters similar to the double-stranded type I fibrils, elongated more slowly and lacked the distinct fibril types seen in fibrils seeded from synthetic seeds (Figure 6). This observation may reflect a greater degree of conformational restraint imposed by the prion seed corresponding to a smaller structural diversity in prions when compared to other PrP amyloid fibrils.^6,7,34^ It is tempting to speculate that PrP amyloid growth might compete with prion replication *in vivo* and that here the kinetics would favor amyloid formation, whereas templating of prions might produce more stable but slower growing fibrils. The observation that structurally distinct PrP fibrils coexisted and grew *in vitro* suggests that fibril populations exist as quasispecies.^3,47^

**Figure 7:**
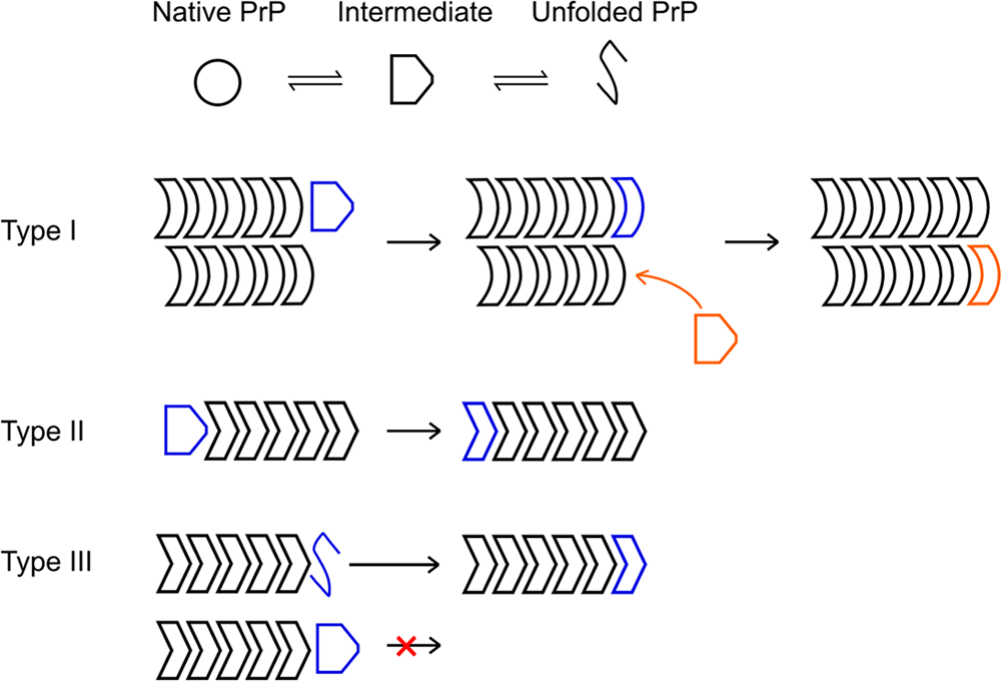
Schematic representation of the elongation mechanisms of fibril types I, II, and III.

Different brightness and growth rates corresponded not only to single and double-strand fibrils but also to different assembly mechanisms, in which type III fibrils grew by addition of unfolded monomers, while types I and II likely incorporated fully or partially folded PrP molecules. (Figure 7). The reduction in type III growth rate at PrP concentrations >5 μM suggests an inhibition of fibril growth by natively folded monomer, similar to the model formulated by Honda et al.^10^ Temperature dependence of type III fibril growth followed Arrhenius’ law. The activation energy of 70 ± 2 kJ/mol is consistent with values calculated for the elongation of Mo PrP 89–230 fibrils in bulk seeding assays,^11^ with *E*_a_ ≈ 170 kJ/mol under conditions where native-state PrP dominated, and *E*_a_ ≈ 50 kJ/mol when PrP was predominantly unfolded.

Low PrP concentration favored type III fibrils; no type I fibril formation was observed below 0.5 μM, which could mean that type I fibrils have a critical concentration above 0.5 μM. Alternatively, monomer addition to double-stranded type I fibrils could follow higher reaction order with the simultaneous incorporation of monomers into both fibril strands. However, the low concentration dependence of type I growth argues against a higher-order mechanism.

The unstructured N-terminal region (NTR) of PrP is not incorporated into the proteinase-K resistant amyloid core of the prion fibril and not necessary for prion replication.^4,48^ Conversely, octarepeat expansions in the NTR of PrP cause genetic prion disease,^49^ and the presence of the NTR facilitates the formation of β-sheet rich oligomeric assemblies,^50^ suggesting a mechanistic role of the NTR in prion assembly. However, the presence of the NTR (PrP 23–230) strongly retarded fibril elongation under our reaction conditions, making further mechanistic analysis unfeasible.

It is commonly observed that fibril elongation retains the structure of the seed,^17^ while the environment can steer fibril populations toward amyloid with different structures and stabilities. Aβ 40 can form multiple stable amyloid structures *in vitro*, whose populations depend on metal ions, pH, temperature, and the presence of cofactors.^17,51,52^ Salt concentration directs α-synuclein into two distinct fibril morphologies, which can propagate faithfully.^53^ Full-length hamster PrP formed two distinct fibril types under different shaking modes,^54^ which differed in their morphologies, internal structures, and ThT fluorescence. The fibril type was maintained after seeding even under unfavorable shaking modes, similar to what was observed in our experiments.

In our study, the structurally distinct PrP fibrils faithfully templated their structures during elongation, while at the same time competing for monomer addition (Figure 4). Monomers adopting an existing template structure is energetically favorable to a change in structure, leading to faithful elongation, even though the seed structure might not be the most stable one under the specific condition.^55^ Environment conditions altered the relative population of propagating species rather than their structures. However, a change in fibril type during growth was occasionally observed, suggesting the fibril end could adopt a limited number of possible conformations, which could interconvert under rare conditions.^56^

In our surface-based elongation experiment, elongation was the dominant, if not only process observed. Fibril elongation was also dominant in previous studies of multiple amyloidogenic proteins, such as Aβ40,^57^ Aβ42,^26^ α-Syn,^25^ and Sup35,^58,59^ but in these systems, no competing fibril species were observed. Under our conditions, we observed no fibril branching^60^ or fragmentation.^28^ Fibrils that appeared where no seed was present in the middle of the measurement could be an indicator of secondary nucleation on the glass surface;^61^ however, the low number of those fibrils suggests that secondary nucleation is not favored. This suggests that our reaction conditions did not favor secondary nucleation or fragmentation, thus simplifying the kinetic analysis.

Elongation of all three fibril types followed a “stop-and-go” pattern over multiple time scales (Figure 2A), as observed for other amyloid proteins.^25,26^ Intermittent growth suggested binding of PrP species that were not able to elongate, or trapping of the bound monomer in an incorrect conformation.^33^ While we cannot exclude that the glass surface contributed to fibril stalling, it is unlikely to be the dominant cause because stall percentages depended on monomer concentration and solution conditions, whereas stalling due to surface interaction and steric hindrance would be expected to be independent of PrP concentration. Similarly, differences in fibril growth are unlikely to be caused by fibril orientation on the surface. If this were the case, we would expect ratios of type I, II, and III fibrils to be independent of monomer concentration and solution condition, which is contrary to our observations.

Similar to other amyloid fibrils,^26^ elongation of synthetic PrP seeds was highly directional. The surfaces of the two fibril ends of a single fibril are not symmetric,^6−8^ which likely results in a slow and a fast interface for fibril growth. We show that in bidirectional fibrils, type II likely represent the slow-growing end and type III the fast-growing end of the same fibril (Figure 2E,F). Our data suggest that RML seeds templated two different fibril types, representing fast and continuous growing fibrils and relatively slow fibrils, which had longer stalls (Figure 6), which may correspond to single and double-strand fibril elongation, respectively. Cryo-EM revealed that 10% of RML prion fibrils were double-stranded.^6^ Unlike most amyloids, a fraction of these double-stranded fibrils had mirror symmetry, indicating an antiparallel orientation of strands and symmetric fibril ends.^6^ Notably, fibrils extending from both sides of a seed were observed in some RML-seeded fibrils (Figure 6A,G, arrows), which could reflect this seed structure. However, the absence of glycosylation in the PrP substrate, the absence of the N-terminal domain of PrP, and the denaturing buffer conditions may have altered fibril structure and elongation kinetics when compared to prion replication *in vivo*.

## Conclusions

Our analysis reveals that, contrary to the long-held assumptions on amyloid growth, polymorphic populations of PrP fibrils coexist and compete under homogeneous conditions, which were previously hidden in ensemble experiments of amyloid kinetics. Our analysis shows that the replication environment shapes the equilibrium between competing isomorphs as posited in the “prion cloud” hypothesis. Our data suggest that prions exist in the form of quasispecies rather than a single isomorphic structure and that rare conformational switching events allow a fibril to “mutate”. Our data therefore support the idea that prions and other amyloid fibrils that replicate by prion-like mechanisms, while nongenetic in nature, satisfy the requirements of molecular evolution by Darwinian principles: metabolism, self-reproduction, and mutability.^15,47^ Prions are inherently able to self-reproduce, at least as long as the underlying polypeptide substrate is able to form amyloid. Mismatch in templating and, possibly, secondary nucleation, generates conformational “mutants”, while the differential in stability between the native and amyloid fold provides the driving energy for replication (“metabolism”). However, it remains to be determined whether prions, analogous to viruses, require external cellular machinery for replication *in vivo*, such as fibril fragmentation by the Hsp104 chaperone in yeast.^62^ Structural and dynamic polymorphism may also underlie competition of infectious prions with noninfectious PrP amyloid *in vivo*. Single-particle analysis permits the dynamic equilibrium between these species to be mapped, which allows us to better understand the intricate balance of protein misfolding, amyloid formation, and prion replication and which can inform more specific therapeutic interventions thattake advantage of the principles of molecular evolution.

## Methods

### Generation of Seeds for Elongation Assay

Frozen aliquots of monomeric PrP were thawed and diluted into buffer to a final composition of 10 μM rPrP in aggregation buffer (50 mM Na-phosphate pH 7.4, 2 M GdnHCl, 300 mM NaCl) with 20 μM ThT. The protein solution was pipetted into a 96-well plate (Corning 3651) with 3 Zirconium (Zr) spheres inside each well. The plate was sealed and inserted into the plate reader (BMG Clariostar). Spontaneous aggregation was initiated by shaking the plate at 700 rpm with 100 s on/20 s off cycles at 42 °C. ThT fluorescence was recorded every 10 min to monitor aggregation kinetics. The assay was stopped at 68 h when the kinetics of selected wells had reached plateau. Selected samples were collected.

Subsequently, a seeding assay was conducted, with the end product of the previous assay as seeds. Seed solution was prediluted 1:10, and sonicated for 10 min in a water bath sonicator. Then seed was added at 0.1% to protein solution whose composition was the same as the previous assay. Seeded aggregation was conducted under the same condition for spontaneous assay. Aggregates were collected after 2 days, aliquoted, and used as seeds for elongation assays.

### Single-Particle Elongation Experiments

An 8-well microscope chamber was washed with Hellmanex II detergent and was plasma cleaned. Diluted seeds were incubated on the coverslip surface for 30 to 45 s, allowing the seed particles to deposit onto the surface. After removing the excess seed, 200 μL monomeric PrP solution at a desired composition with 400 nM Nile Blue was added. The chamber was then sealed and ready to be imaged by the Nikon, Eclipse Ti2-E inverted microscope.

The temperature was maintained by an incubator box around the microscope body. For time-lapse imaging, MetaMorph imaging software was used to automatically take images of multiple field of views (usually >10) initially selected by users at a 15 min interval, for a total of >2 days.

For data analysis, images taken at each location were imported into ImageJ as a time-lapsed image stack. The image stack was drift corrected by ImageJ plugin StackReg^63^ or Image Stabilizer.^64^ Usually, 3–4 stacks were analyzed, and in total >70 fibrils that met our selection criteria were analyzed for each set of conditions.

To extract kinetic information, a kymograph was generated for each fibril in ImageJ and the fibril edge was selected manually by drawing a segmented line. The saved fibril edge positions were analyzed using custom scripts written in MATLAB, to identify the growth/stall phase, calculate the overall rate (final length/total time), pause free rate (final length/time spent in the growing phase), and stall percentage and other parameters.

### Sequential Seeding Assay

A sequential seeding assay of MoPrP 91 was performed in buffer containing either 1 M (condition a) or 2 M GdnHCl (condition b), plus 50 mM Na-phosphate pH 7.4, 300 mM NaCl. Solution monomer concentration was 1 μM and seed concentration was 0.1% (w/w) with respect to monomer.

In the first seeding assay, the mixed solution with seed, monomer in the specific buffer was pipetted into plate wells with 3 Zr beads in each well, and incubated in BMG plate reader at 37 °C with agitation. The seeded products were labeled based on their buffer conditions, a or b, and were used as seeds for the second assay.

In the second assay, the use of two seed types (seed a and b) and two solution conditions (buffer a and b) generated four experiment combinations and four end products: aa, ab, ba, and bb. Here the first letter represented the seed type (i.e., the buffer type of the first seeding assay), and the second letter represented the buffer condition of the second assay.

## Supporting Information Available

Supplementary methods, figures including structures of PrP seed and elongated fibrils, analysis of elongation kinetics under different conditions (PDF)
Video showing seed elongation (MP4)
Video showing seed elongation (MP4)

## Abbreviations

CD: circular dichroism
FOV: field of view
MoPrP: mouse prion protein
NR: Nile Red
NB: Nile Blue
PK: Proteinase K
PrP: prion protein
PrP^C^: cellular prion protein
TAB: transient amyloid binding
TIRFM: total internal reflection microscopy
